# Mediators of Monocyte Migration in Response to Recovery Modalities following Resistance Exercise

**DOI:** 10.1155/2014/145817

**Published:** 2014-06-02

**Authors:** Adam R. Jajtner, Maren S. Fragala, Jeremy R. Townsend, Adam M. Gonzalez, Adam J. Wells, David H. Fukuda, Jeffrey R. Stout, Jay R. Hoffman

**Affiliations:** Institute of Exercise Physiology and Wellness, University of Central Florida, Orlando, FL, USA

## Abstract

Mediators of monocyte migration, complement receptor-3 (CR3), and chemokine ligand-4 (CCL4) were measured in response to recovery modalities following resistance exercise. Thirty resistance-trained men (23.1 ± 2.9 y; 175.2 ± 7.1 cm; 82.1 ± 8.4 kg) were given neuromuscular electric stimulation (NMES), cold water immersion (CWI), or control (CON) treatments immediately following resistance exercise. Blood samples were obtained preexercise (PRE), immediately (IP), 30 minutes (30 P), 24 hours (24 H), and 48 hours (48 H) after exercise for measurement of circulating CCL4 and CR3 expression on CD14+ monocytes, by assay and flow cytometry. Circulating CCL4 showed no consistent changes. Inferential analysis indicated that CR3 expression was likely greater in CON at 30 P than NMES (90.0%) or CWI (86.8%). NMES was likely lower than CON at 24 H (92.9%) and very likely lower at 48 H (98.7%). Expression of CR3 following CWI was very likely greater than CON (96.5%) at 24 H. The proportion of CR3+ monocytes was likely greater following CWI than NMES (85.8%) or CON (85.2%) at 24 H. The change in proportion of CR3+ monocytes was likely (86.4%) greater following NMES than CON from IP to 30 P. The increased expression of CR3 and increased proportion of CR3+ monocytes following CWI at 24 H indicate a potentially improved ability for monocyte adhesion to the endothelium, possibly improving phagocytosis of damaged tissues.

## 1. Introduction

Skeletal muscle damage resulting from resistance exercise stimulates an inflammatory cascade initiated by cytokines [1]. In addition to the increases in markers of muscle damage like creatine kinase (CK) and myoglobin, the inflammatory responses to resistance exercise includes, among many others, increases in tumor necrosis factor-*α* (TNF-*α*), interleukin-10 (IL-10), and C-reactive protein (CRP) [1–4]. During the inflammatory process, the cell adhesion molecule complement receptor 3 (CR3), consisting of the integrin molecules cluster of differentiation (CD)11b and CD18 [5], facilitates monocyte vascular adhesion and migration into the damaged tissue, thereby promoting phagocytosis of opsonized particles [5, 6]. Prior to extravasation, monocytes are signaled to the site of inflammation, primarily through chemotaxis [7]. During chemotaxis, several chemokines are secreted, including macrophage inflammatory protein-1*β*, also known as chemokine ligand 4 (CCL4) [8]. While the actions of CCL4 and CR3 on leukocytes are not fully understood, previous research has shown increases in CCL4 concentration [9] and CR3 expression following exercise [10]. Many other studies, however, have found equivocal results [11–13]. Additionally, whether these processes can be altered by athletic training therapies following muscle damaging exercise is presently unknown.

Cold water immersion (CWI) therapy is a common recovery modality utilized to assist athletes in recovery from high intensity exercise [14]. CWI can alleviate delayed onset muscle soreness (DOMS) and reduce the efflux of creatine kinase (CK) up to 48 hours following exercise [14]. CWI has also been demonstrated to decrease postexercise leukocytosis or increase the number of leukocytes, specifically, polymorphonuclear cells and lymphocytes, in response to muscle damage [15, 16]. CWI has been demonstrated to modulate cytokine and chemokine activity [4, 17], while cold exposure* in vitro* has shown to increase the expression of cell adhesion molecules, specifically the *β*2-integrin CD11b/CD18, on cultured neutrophils [18], which may mediate phagocytic migration [5]. However,* in vivo* examination of the influence of CWI on the processes of monocyte cell adhesion is yet to be demonstrated.

Neuromuscular electrical stimulation (NMES) is another recovery modality intended to increase blood flow to the inflamed tissue [19, 20]. Despite the minimal benefits to strength and power recovery [19, 21], NMES may be beneficial for removal of metabolic waste [22] and reducing circulating levels of CK [21]. Recent work from our lab has indicated that the use of NMES suppressed the natural increase in TNF-*α* receptor-1 on monocytes 30 minutes after exercise which remained for 48 hours following an acute heavy resistance training bout [3], indicating a modulation of the immune response. However, how NMES potentially intervenes during the postexercise monocyte cell adhesion cascade is yet to be determined.

Investigation of the expression of CR3 on monocytes and concomitant circulating concentrations of CCL4 during recovery from muscle damaging exercise will help us to better understand the processes and mechanisms of exercise-induced muscle damage and subsequent repair. Additionally, examining how recovery modalities mediate the cell adhesion response may provide specific evidence supporting various strategies used to facilitate recovery. To our knowledge, this will be the first study to examine the impact of a dynamic strength training session on CCL4 and CR3. Therefore, the purpose of this study was twofold. First, we examined the circulating CCL4 and CR3 responses to acute high volume resistance exercise. Secondly, we examined the influence of two recovery methods (NMES and CWI) following resistance exercise on the concentration of the chemokine CCL4 and expression and proportion of CR3+ cells.

## 2. Methods

### 2.1. Participants

Thirty resistance-trained males volunteered to participate in this study. Participants were randomly divided into three treatment groups: control (CON) (*n* = 10), neuromuscular electrical stimulation (NMES) (*n* = 10), and cold water immersion (CWI) (*n* = 10). Subjects' characteristics are depicted in Table 1. Following an explanation of all procedures, risks, and benefits, each participant gave his written informed consent prior to participation in this study. The Institutional Review Board of the university approved the research protocol. For inclusion in the study, participants had to have a minimum of one year of resistance training experience, particularly in the squat exercise. Participants were not permitted to use any additional nutritional supplements or medications while enrolled in the study. Screening for nutritional supplements and performance enhancing drug use was accomplished via a health history questionnaire completed during participant recruitment. Participants were instructed not to partake in any additional recovery strategies while enrolled in the study including NSAIDs, saunas, stretching routines, foam rollers, massages, and additional hot/cold water therapy.

### 2.2. Study Protocol

Participants reported to the Human Performance Laboratory (HPL) on four separate occasions. On the first visit (T1), participants were tested for maximal strength (1-RM) on the barbell back squat, dead lift, and barbell split squat exercises. Prior to the second visit (T2), which occurred at least 72 hours after T1, participants were instructed to refrain from all forms of exercise for a minimum of 72 hours. Also, prior to subsequent exercise sessions, participants were instructed to report to the HPL in a 10-hour fasted state. During T2, participants performed a lower body resistance exercise session which consisted of four sets of the squat, dead lift, and barbell split squat exercises. The rest interval between each set and between all exercises was 90 s. The squat exercise was performed with 80% of the participant's previously measured 1-RM, while the dead lift and barbell split squat exercises were performed with 70% of the participant's previously measured 1-RM. Participants were encouraged to perform as many repetitions as possible, but not to exceed 10 repetitions in any set. This protocol was utilized to simulate a typical high intensity lower body training routine during a hypertrophy phase of training. Participants then reported back to the HPL 24 (T3) and 48 hours (T4) after exercise. During T3 and T4, participants performed four sets of the squat exercise only using the same loading pattern and rest interval length as T2 to monitor strength and power. Participants were asked to complete dietary logs during the two days of recovery. Blood samples were obtained at five time points over the course of the study: baseline (BL), immediately after exercise (IP), and 30 min after exercise (30 P) during T2 and 24 (prior to exercise on T3) and 48 hours (prior to exercise on T4) after T2 (24 H and 48 H, resp.). All trials started between 7 and 9 AM with each individual starting at the same time of day. This allowed blood samples obtained prior to exercise to be collected within 15 minutes of the previous day.

### 2.3. Neuromuscular Electrical Stimulation Therapy (NMES)

Participants assigned to NMES were provided with 24 minutes of electrical stimulation immediately following the postexercise blood draw (T2) or after exercise (T3) using a commercially available product. All treatments were provided according to the manufacturer's instructions (Compex Performance US, DJO, Vista, CA). Briefly, the participant was placed in a supine position with three electrodes placed on each of the quadriceps. Specifically, one large electrode with a negative charge was placed at the most proximal point of the upper leg, while two small electrodes with positive charge were placed on the belly of the vastus lateralis and vastus medialis. The unit was set to an established recovery mode, while the participants were asked to increase the stimulation intensity to the highest possible level without undue discomfort. The research team observed the participants during stimulation to assist with proper intensity of stimulation while also recording the stimulation setting as previously noted [23]. The treatment protocol consisted of nine sequences, with the first three stages lasting for two minutes and the remaining six for three minutes. Frequency of contraction started at 9 Hz, stepping down 1 Hz per stage to 1 Hz. The participants were asked to remain in a supine position throughout the 24 minutes of treatment.

### 2.4. Cold Water Immersion (CWI)

The participants in CWI were required to fully immerse their lower body into a metal tub (58.4 cm × 121.9 cm) filled to 22.9 cm of height with ice water at 10°–12°C immediately following postexercise blood draw (T2) or immediately after exercise (T3). Participants sat in the water up to their umbilicus for 10 min. Once the participants completed the 10 minutes in the ice bath they were asked to remain in the HPL for an additional 20 minutes to ensure a similar postexercise intervention opportunity with NMES and CON, who remained in the HPL for 30 minutes in a supine position.

### 2.5. Blood Measurements

During T2, PRE blood samples were obtained following a 15 min equilibration period. Additional blood samples were also drawn at IP and 30 P. All blood samples were obtained using a Teflon cannula placed in a superficial forearm vein using a three-way stopcock with a male luer lock adapter. The cannula was maintained patent using an isotonic saline solution (Becton Dickinson, Franklin Lakes, NJ). IP blood samples were taken within 1 minute of exercise cessation. Following the resistance exercise protocol, participants remained in the supine position for the full 30 min recovery phase prior to the 30 P blood sample being drawn, except for the participants in the CWI groups, who spent the first 10 minutes of the 30 minutes in the ice bath. All T2 blood samples were drawn with a plastic syringe while the participant was in a supine position. During T3 and T4, only preexercise blood samples were drawn (24 H and 48 H, resp.) following a 15 min equilibration period. These blood samples were obtained from an antecubital arm vein using a 20-gauge disposable needle equipped with a Vacutainer tube holder (Becton Dickinson, Franklin Lakes, NJ). Blood samples of each participant were obtained at the same time of day during each session.

All blood samples were collected into two Vacutainer tubes, one containing no anticlotting agent and the second containing K_2_EDTA. A small aliquot of whole blood was removed from the second tube and used for determination of hematocrit and hemoglobin. The blood in the first tube was allowed to clot at room temperature for 30 minutes and subsequently centrifuged at 3,000 ×g for 15 min along with the remaining whole blood from the second tube. The resulting plasma and serum were placed into separate 1.6-mL microcentrifuge tubes and frozen at −80°C for later analysis.

### 2.6. Cell Staining

Samples were obtained from fresh, anticoagulated (K_2_EDTA) whole blood and analyzed in duplicate. Erythrocytes were first lysed from 350 *μ*L of whole blood with BD Pharm Lyse solution (BD Biosciences, Franklin Lakes, NJ) within 30 min of collection. Samples were then washed in staining buffer containing 1 x phosphate-buffered saline containing fetal bovine serum (BD Pharminigen Stain Buffer; BD Biosciences) by centrifugation and aspiration three times. Leukocytes were then resuspended in 100 *μ*L BD Pharminigen Stain Buffer. Direct staining methods were used to label CR3 and CD14. Allophycocyanin (APC) conjugated anti-CR3 (D12; BD Pharminigen) and PerCP Cy5.5 conjugated anti-CD14 (M5E2; BD Pharminigen) were used in the receptor labeling process. Surface staining was performed by adding 20 *μ*L of directly conjugated APC-anti-CR3 and 5 *μ*L of directly conjugated PerCP Cy5.5-anti-CD 14 to the cell suspension and incubating in the dark for 30 min at 20°C. Cells were resuspended in 1.0 mL of stain buffer for flow cytometry analysis.

### 2.7. Flow Cytometry

**Figure 1:**
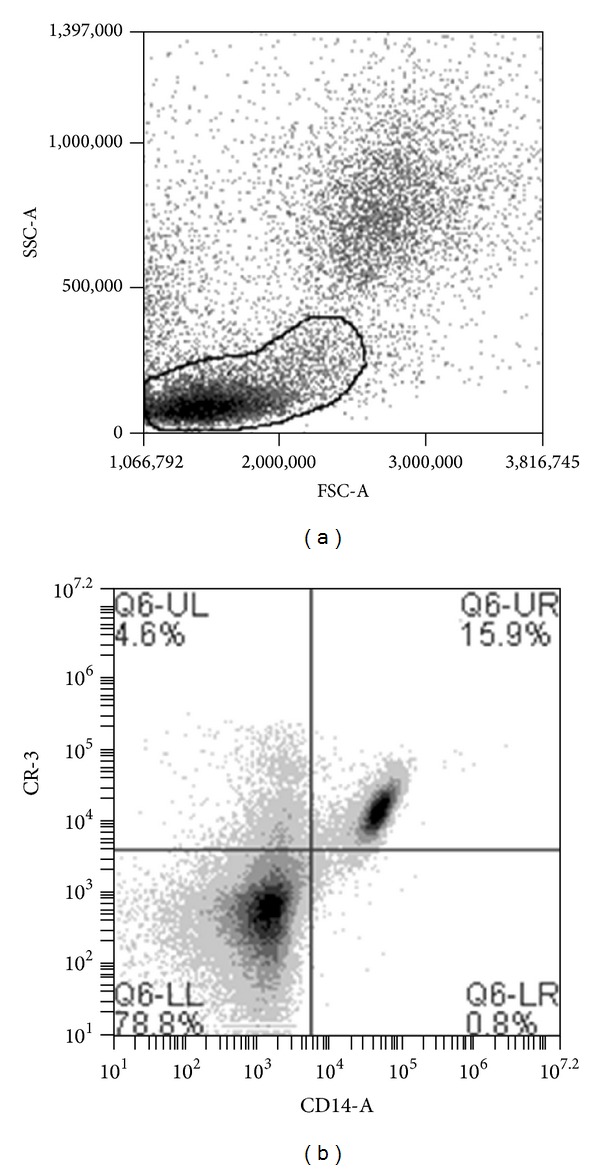
Gating procedures for CD14+ cells. (a) CD14+ cells selected from the specified region based on forward (FSC) and side scatter (SSC) properties. (b) Two-dimensional histogram displaying fluorescence characteristics of cells in selected region. Cells positive for CR3 and CD14 are displayed in the upper right quadrant.

Flow cytometry analysis of stained cells was run on a BD C6 Accuri Flow Cytometer (BD Biosciences, San Jose, CA), equipped with BD Accuri analysis software (BD Biosciences). Forward and side scatter along with two fluorescent channels of data were collected using two lasers providing excitation at 488 and 640 nm. Monocytes were determined by initial gating based on forward and side scatter, followed by gating for CD14^+^ cells as also described by Tallone and colleagues [24]. A minimum of 10,000 events, defined as CD14^+^ monocytes, was obtained with each sample (Figure 1).

Analysis of monocyte subpopulations was completed by quadrant analyses, in which CD14 was compared with CR3. Mean fluorescence of CR3 on CD14+ cells was recorded, representing the expression of CR3 per cell [25]. Proportion of CR3+/CD14+ versus CR3−/CD14+ were determined by quadrant analysis (Figure 1). Compensation for fluorescence spillover was set based on manufacturer recommendations (BD Biosciences).

### 2.8. Biochemical Analysis

Circulating levels of chemokine (C-C motif) ligand 4 (CCL4), also referred to as macrophage inhibiting protein-1*β*, were assessed by Magpix (EMD Millipore, Billerica, MA, USA) via the human cytokine/chemokine panel one (EMD Millipore, Billerica, MA, USA). Samples were analyzed according to manufacturer's guidelines with an average coefficient of variation 6.25%.

Creatine kinase (CK) was analyzed with the use of a spectrophotometer and a commercially available enzymatic kit (Sekisui Diagnostics, Charlottetown, PE, Canada) per manufacturer's instructions. Determination of serum immunoreactivity values was determined using a BioTek Eon spectrophotometer (BioTek, Winooski, VT, USA). To eliminate interassay variance, all samples for a particular assay were thawed once and analyzed in the same assay run by a single technician. All samples were run in duplicate with a mean intra-assay variance of 2.99%.

### 2.9. Dietary Logs

Participants were instructed to record as accurately as possible everything they consumed during workout days T2 and T3. Participants were instructed not to eat or drink (except water) within 10 hours of reporting to the HPL for subsequent visits. FoodWorks Dietary Analysis software (McGraw Hill, New York, NY) was used to analyze the dietary recalls for total kilocalorie intake (kcal) and macronutrient distributions (carbohydrate, protein, and fat).

### 2.10. Statistical Analysis

All biochemical changes were analyzed using a repeated measures analysis of variance (ANOVA). Pairwise comparisons with the Bonferroni adjustment were employed for significant main effects, while a Tukey* post hoc* test was employed for significant interactions. Prior to analysis all data were assessed to ensure normal distribution, homogeneity of variance, and sphericity. Changes in dietary composition were analyzed using repeated measures ANOVA. Results were considered significant at an alpha level of *P* ≤ 0.05. All data are reported as mean ± SD.

The effects of treatment modalities were analyzed using magnitude-based inferences calculated from 90% confidence intervals, as previously described by Batterham and Hopkins [26]. Magnitude based inferences are a statistical approach to data analysis which uses within-subject modeling as opposed to combining data into a single unit of change as with repeated measures. Magnitude based inferences have also been used previously in conjunction with null hypothesis testing [2, 4, 27] to allow for more sensitive interpretation, providing more practically applicable understandings of the results. Additionally, magnitude based inferences help determine whether a significant *P* value is meaningful, as significant *P* values have been determined to be trivial previously [27]. Absolute values and changes from IP were analyzed to assess differences between groups at each individual time point and across time, respectively. The IP time point was selected as the initial baseline as no recovery methods had been employed prior to IP. These values were then analyzed via a published spreadsheet [28], with the smallest nontrivial change set at 20% of the grand standard deviation [26]. All data are expressed with percent chances of a beneficial, trivial, and negative outcome. Qualitative inferences, based on quantitative chances were assessed as <1% almost certainly not, 1–5% very unlikely, 5–25% unlikely, 25–75% possibly, 75–95% likely, 95–99% very likely, and >99% almost certainly [29].

## 3. Results

Groups did not differ in baseline physical characteristics. Average resistance training experience was 6.5 ± 3.5 years, and participants had an average squat 1RM of 151.0 ± 31.0 kg. No group differences were noted in the quantity of repetitions performed, markers of muscle damage (CK) 24 and 48 hours after exercise, or macronutrient composition on T2 and T3. Decreases in repetitions from T2 (23.1–27.9) were noted for all groups combined at T3 (−30%; 14.1–20.9) and T4 (−24%; 15.9–21.8). Additionally, increases in CK concentrations from PRE (103.4–166.3 U/L) were observed at 24 H (290%; 468.9–677.1 U/L) and 48 H (272%; 424.6–612.5 U/L). Performance and nutritional data along with plasma volume shifts have been previously reported [2]. Results have not been corrected for plasma volume shifts.

### 3.1. CCL4 Concentration

**Figure 2:**
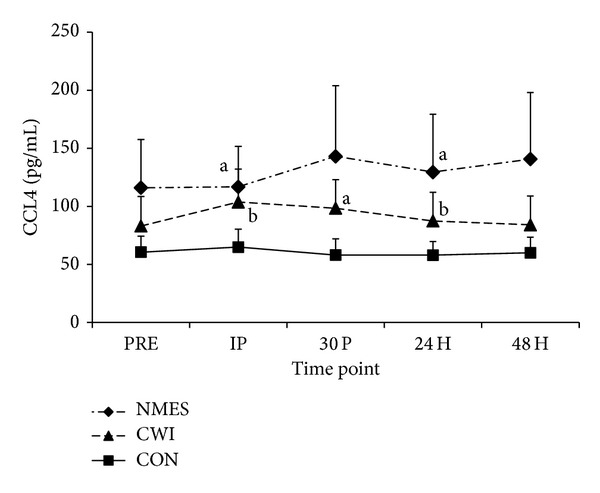
Circulating Concentration of CCL4 (mean ± SE). Changes in CCL4 concentration before exercise (PRE), immediately after exercise (IP), and 30 minutes (30 P), 24 hours (24 H), and 48 hours (48 H) after exercise in neuromuscular electrical stimulation (NMES), cold water immersion (CWI), and control (CON) groups. (a) The value is “likely” greater than CON at the specified time point. (b) The value is “possibly” greater than CON at the specified time point.

No significant difference occurred between groups at IP (*P* = 0.393). Additionally, no significant main effect for time (*P* = 0.317) was observed. Magnitude based inferences indicate that NMES was likely greater than CON at IP (percent chance: 80.4%) and 24 H (84.3%). CWI was possibly greater than CON at IP (73.2%) and 24 H (65.4%), while it was likely greater (77.7%) at 30 P. When the change of values was assessed, the changes from IP were possibly increased in NMES versus CON at 30 P (70.0%), 24 H (52.5%), and 48 H (65.6%). Additionally, changes from IP were possibly increased in NMES compared to CWI at 24 H (70.7%) and likely greater at 48 H (82.7%). Changes from IP to 24 H and 48 H were likely (83.6%) and possibly (64.7%) trivial, respectively, when comparing CON to CWI (Figure 2).

### 3.2. CR3 Expression (Reported as Mean Fluorescence) on CD14+ Monocytes

**Figure 3:**
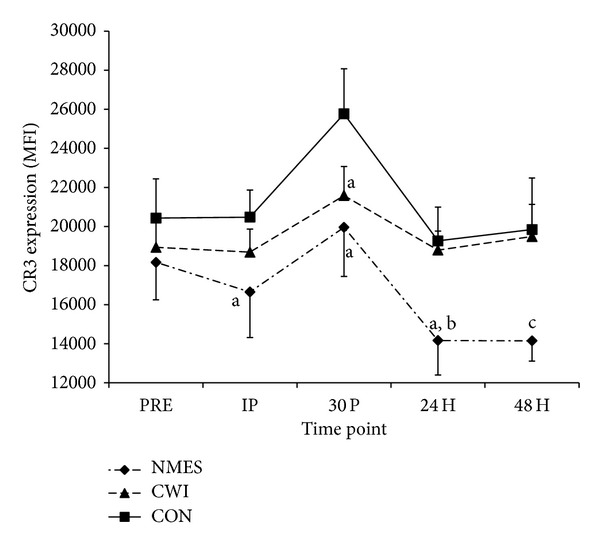
Expression of the complement receptor 3 (CR3) on CD14+ monocytes (mean ± SE). CR3 expression, as measured by mean fluorescence intensity before exercise (PRE), immediately after exercise (IP), and 30 minutes (30 P), 24 hours (24 H), and 48 hours (48 H) after exercise in neuromuscular electrical stimulation (NMES), cold water immersion (CWI), and control (CON) groups. (a) The value is “likely” lower than CON at the specified time point, (b) the value is “very likely” lower than CON at the specified time point, and (c) the value is “very likely” lower than CWI at the specified time point.

Mean fluorescence intensity on CD14+ monocytes showed that no significant differences were observed between groups at IP (*P* = 0.269); however, there was a significant main effect for time (*P* = 0.004). Pairwise comparisons indicate that CR3 was elevated at 30 P compared to PRE (*P* = 0.015), IP (*P* = 0.005), and 24 H (*P* = 0.005) (Figure 3).

Magnitude based inferences indicate that CR3 fluorescence on CD14+ monocytes was likely lower following NMES than CON at IP (85.7%), 30 P (90.0%), and 24 H (92.9%), while it was very likely lower at 48 H (98.7%). Additionally, mean fluorescence following CWI was likely lower (86.8%) than CON at 30 P, while it was very likely greater than NMES at 24 H (96.5%). Changes in CR3 expression on CD14+ monocytes were unclear for all time points and recovery modalities (Figure 3).

### 3.3. Proportion of CR3+ Monocytes (CR3+/CD14+)

**Figure 4:**
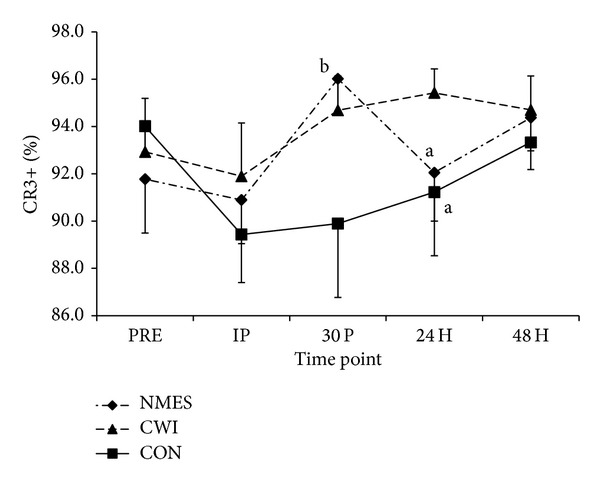
Proportion of CR3+/CD14+ monocytes (mean ± SE). Changes in CR3 expression before exercise (PRE), immediately after exercise (IP), and 30 minutes (30 P), 24 hours (24 H), and 48 hours (48 H) after exercise in neuromuscular electrical stimulation (NMES), cold water immersion (CWI), and control (CON) groups. (a) A “likely” lower value than CWI at the specified time point. (b) A “likely” greater change from IP than CON at the specified time point.

No difference between groups at IP was observed (*P* = 0.690). Additionally, only a trend for a main effect of time (*P* = 0.074) was observed. Magnitude based inferences indicate that CWI resulted in a likely greater proportion of CR3+ monocytes than NMES (85.8%) and CON (85.2%) at 24 H (Figure 4). Additionally, when changes from IP were analyzed, the increase from IP to 30 P in proportion of CR3+ monocytes was likely (86.4%) greater in NMES than CON while all other analyses were unclear.

## 4. Discussion

The main findings of this investigation indicate that mean florescence intensity expression of CR3 on CD14+ monocytes was elevated following an acute bout of heavy resistance exercise. Recovery treatments appeared to affect both the expression of CR3 and proportion of monocytes expressing CR3. Postexercise treatments with NMES and CWI appeared to attenuate the increased expression of CR3 observed in CON. Additionally, NMES appears to reduce expression of CR3 on CD14+ monocytes in comparison to both CWI and CON at 24 and 48 hours after exercise. Conversely, NMES likely increased the proportion of circulating CD14+ monocytes expressing CR3 immediately following treatment compared to the control group. During recovery, CWI likely resulted in a higher proportion of circulating CD14+ monocytes expressing CR3 at 24 H. Circulating CCL4 showed large interindividual variation and did not appear to be consistently affected by the exercise protocol or treatments.

As CCL4 is a chemotactic cytokine responsible for the recruitment of monocytes, natural killer cells, and other immune cells, we had hypothesized that the muscle damaging exercise would have resulted in significant elevations in CCL4 as part of the cell mobilization process. Similarly, we expected changes in CCL4 response as a result of blood flow alterations induced by the treatment modalities. However, contrary to our hypothesis, circulating CCL4 did not appear to be strongly influenced by exercise or treatment. While previous research has shown circulating CCL4 to be elevated in response to cardiovascular exercise [9], investigations using resistance exercise similarly found no changes in circulating CCL4 following eccentric leg extensions [11] and 3 × 6 RM of the squat, front squat, and leg extension exercise [13]. CCL4 production is stimulated by proinflammatory cytokines (e.g., TNF*α*, IFN*γ*, and IL-1*β*), while at the same time inhibited by anti-inflammatory cytokines (e.g., IL-10) [8]. Since we previously reported that TNF*α* and IL-10 were increased 30 minutes following this resistance exercise protocol [2, 4], it is plausible that the interaction between these pro- and anti-inflammatory factors may initiate competing signals leading to the unclear result observed in this study. However, the large interindividual variation in circulating CCL4 observed in our study may have also masked any group trends in the data. As CCL4 is a chemokine secreted by most immune cells [8], large differences between individuals are expected and have been previously demonstrated [30]. Future research should further investigate the time course of CCL4 concentration following resistance exercise and how it relates with pro- and anti-inflammatory factors such as TNF*α* and IL-10.

CR3 expression significantly increased 30 minutes following heavy resistance exercise, which returned to baseline levels approximately 24 hours later. Previous studies examining CR3 expression on monocytes in response to different exercise protocols have found no increase in receptor expression immediately following exercise [10, 12] but have found increases during the recovery period [10]. Nevertheless, the timing of the expression of CR3 on circulating monocytes appears to be controversial [10, 12]. As monocytes are a primary source of proinflammatory cytokines following exercise [1], their role in the subsequent inflammatory response is essential. Typically, granulocytes migrate to the site of inflammation within hours, while macrophages are observed to increase in damaged tissue 24 hours following exercise [31–34]. Monocytes are signaled to the site of inflammation by chemotaxis and mediated by chemokines [7], which have been demonstrated to increase following resistance or aerobic exercise within one hour [9, 35], promoting the monocyte response. One potential influence on CR3 expression is training status [36]. The resistance-trained individuals recruited to participate in the present study may have different response patterns to the training stimulus as compared to nontrained or recreationally active individuals used in previous investigations [10, 12]. It is possible that higher fitness level will attenuate the monocytosis observed following exercise [13]. It is important to consider that although the present study attempted to provide a unique timeline for CR3 expression changes during 48 hours of recovery, changes could have occurred between sampling periods that were not measured. Further studies are needed to identify the time sequence of CR3 expression in trained and untrained populations.

While both recovery modalities utilized in this study appear to blunt the postexercise response in CR3 expression on CD14+ monocytes at 30 P, NMES suppressed CR3 expression on CD14+ monocytes at 24 and 48 hours following the initial exercise stimulus. To the best of our knowledge, no prior studies have examined the expression of CR3* in vivo* or on monocytes following CWI. Nash and colleagues [18], however, have previously demonstrated a gradual increase in expression of CR3 when cultured neutrophils were cooled to 10°C* in vitro*. The contrasting results of this study may be due to a different response in monocytes when cooled. Alternatively, a reduced leukocytosis following CWI* in vivo* [15] may explain the reduced expression of CR3 at 30 P following CWI. During the postexercise period, monocyte influx appears to occur with a high expression of CR3 [13, 16]. Our results indicate that CWI may reduce overall CR3 expression at 30 P by blunting the release of monocytes with elevated CR3 expression. It is important to note, however, that a decreased leukocytosis following CWI is controversial [37], and, to the best of our knowledge, no studies have examined monocyte response following NMES. This emphasizes the need for further research to determine the source and impact of reduced expression of CR3 in monocytes following treatment.

At 24 H, we observed a likely greater proportion of CR3+ monocytes in CWI compared to NMES and CON. While the proportion of CR3+ cells was consistent with previous research [10], the difference observed following treatments is a novel finding. As monocytes typically circulate for several days [38], the increased proportion of CR3+ monocytes observed in this study at 24 H may be a function of increased expression, which may occur via multiple mechanisms. Previously, it has been demonstrated that 20 minutes of exposure to 10°C* in vitro* nearly doubles the expression of CR3 on granulocytes [18]. It is possible that this may also occur with monocytes, leading to the observed increase following CWI. Another potential mechanism is elevation of C-reactive protein concentrations at 24 hours after exercise [2] which has been demonstrated to upregulate CD11b and CD18 [39], which together compose CR3 [5]. Interestingly, the increases in CR3+ monocytes at 24 H observed in this study were not observed at 48 H. While being speculative, it is possible that the increased C-reactive protein may be a more likely explanation as it followed the same time course [2]. As IL-10 increases following cold exposure [2], another potential mechanism would be IL-10 mediated expression as IL-10 expression has been demonstrated to increase CR3 expression in an animal model [40]. Further research is warranted to determine whether a greater proportion of CR3+ cells, or simply CR3 expression, leads to increased extravasation of monocytes and/or phagocytosis.

Additionally, the change in the proportion of CR3+/CD14+ cells compared to CR3−/CD14+ cells from IP to 30 P was likely greater in NMES versus CON. This increase may be indicative of increased extravasation immediately following NMES treatment. As NMES treatment increases blood flow to inflamed tissue [19, 20], observed increases in the proportion of CR3+ cells may be due to blood flow changes. It is also possible that NMES affected proportion of CR3+ cells via cytokine signaling. Recent work from our lab has indicated that the use of NMES attenuated the increase in TNF-*α* receptor 1 and IL-10 30 minutes following exercise [2, 3]. Additionally, IL-10 may affect expression of CR3 on monocytes by way of granulocyte colony stimulating factor (G-CSF) [41, 42]. Future studies are needed to examine the mechanisms of NMES during recovery, as well as CR3 expression at additional time points following the cessation of treatment.

While this is the first study to report changes in adhesion molecules on monocytes following different recovery modalities after exercise induced muscle damage, several considerations should be noted in the interpretation of the results. The first consideration is the training status of the participants. Resistance-trained men were recruited for the study to ensure that participants could complete the strenuous exercise protocol and to avoid potential risks of implementing the protocol to untrained persons. While recruitment criteria required that all participants have a minimum training experience of 1 year, there remains the potential that the relative stress of the protocol varied by individual. Secondly, the limitations of the blood sampling intervals must be considered. Following the 30 P sampling time point, there was almost 24 hours between blood samples. During this 24-hour period, it is possible that inflammation from muscle damage and anti-inflammatory effects of therapeutic modalities may induce changes in cytokine and chemokine concentrations that may influence CCL4 concentration and adhesion molecules [12, 43]. Finally, while this investigation sought to explain mechanisms underlying recovery specific to CR3 and CCL4, it is important to keep in mind that several other inflammatory cells and cytokines are involved in the recovery process which may impact the actions of CD14+ monocytes. Future studies are needed to examine changes in cell counts across more frequent sampling during the recovery period with therapeutic modalities and immune cell measures.

In conclusion, expression of CR3 on CD14+ monocytes was elevated following acute muscle damaging resistance exercise implying an increased potential for each individual cell to adhere to endothelial walls at 30 minutes after exercise. By 24 hours into recovery, the elevated expression of CR3 on CD14+ monocytes had returned to baseline. Treatment with NMES appeared to attenuate the expression of CR3 on CD14+ monocytes during recovery up to 48 hours, indicating a reduced potential for cellular endothelial adherence. Additionally, treatment with CWI likely increased the proportion of CR3+ monocytes. Although we demonstrated exercise and treatment influences on monocyte CR3 expression, the subsequent impact of altered expression of CR3 or proportion of CR3+ cells on extravasation and phagocytosis is not fully understood.

## Figures and Tables

**Table 1:** Subject characteristics.

	NMES	CWI	CON
*n*	10	10	10
Age (y)	23.0 ± 3.0	22.5 ± 3.0	23.8 ± 3.0
Height (cm)	175.9 ± 7.6	171.4 ± 6.6	178.3 ± 5.6
Weight (kg)	83.5 ± 9.8	77.1 ± 7.7	85.7 ± 5.4
BMI	27.0 ± 2.55	26.5 ± 2.13	27.0 ± 2.44

Descriptive characteristics of participants in neuromuscular electrical stimulation (NMES), cold water immersion (CWI), and control (CON).
